# Establishment of national reference for bunyavirus nucleic acid detection kits for diagnosis of SFTS virus

**DOI:** 10.1186/s12985-017-0682-z

**Published:** 2017-02-16

**Authors:** Xu Lu, Ling Wang, Dongting Bai, Yuhua Li

**Affiliations:** Key Laboratory of the Ministry of Health for Research on Quality and Standardization of Biotech Products, National Institutes for Food and Drug Control, Beijing, 100050 People’s Republic of China

**Keywords:** SFTSV, Viral nucleic acid detection kit, Reference, q-PCR

## Abstract

**Background:**

Severe fever with thrombocytopenia syndrome (SFTS) caused by SFTS virus (SFTSV) usually have a high fatality. At present no effective therapy or vaccine are available, so early diagnosis of SFTS is crucial to prevent and control SFTSV infection. This study aimed to establish a national reference for these diagnostic kits of SFTSV genome and make the diagnosis of the disease effective.

**Methods:**

Six SFTSV strains isolated from different regions, and five relative viruses with similar clinical manifestations were selected as positive and negative references and assessed using real time quantitative PCR (q-PCR) using specific primers and probe and two commercial kits. The stability of the references was also assessed at 37 °C, room temperature or -70 °C for 8 days, 14 days or 8 months respectively, or following several cycles of freezing-thawing. Collaborative calibration of the references was performed by three labs.

**Results:**

The references indicated good accuracy and specificity. The lowest detection limit was 10^2^ U/mL. The accuracy was coefficient of variation less than 5%. The references were highly stable at high temperatures and after long storing and freezing-thawing treatment.

**Conclusions:**

We successfully established a national reference with good accuracy, high specificity, sensitivity and stability, which can be applied for quality control of commercial SFTSV diagnostic kits, thus preventing and controlling SFTS.

**Trial registration:**

The references have been finished and it was retrospectively registered in the following article.

**Electronic supplementary material:**

The online version of this article (doi:10.1186/s12985-017-0682-z) contains supplementary material, which is available to authorized users.

## Background

In 2007, some patients with severe fever, gastrointestinal bleeding and thrombocytopenia syndrome were first discovered in central and eastern China [1, 2], but the pathogen cannot be determined. The pathogen responsible for the syndrome was not identified until a novel *Phlebovirus* (family *Bunyaviridae*) named Sever Fever Thrombocytopenia Syndrome virus (SFTSV), was isolated from a sample collected from a patient in Henan Province in China at 2009 [3]. Then the cases were successively reported in Korea and Japan [4–6].

SFTSV, like other viruses in genus *Phlebovirus*, is an enveloped, segmented minus-strand RNA virus. The genome consists of three segments: L (large), M (middle) and S (small), which respectively encode the RNA-dependent RNA polymerase, Gn-Gc envelope glycoproteins, nucleocapsid and non-structural proteins [3].

SFTSV infection is clinically characterized by fever, thrombocytopenia and leukopenia, gastrointestinal symptoms, and multiorgan dysfunction. The initial case fatality of SFTSV infection was 30% [3, 7] in China and according to the national surveillance data, the case fatality from 2011 to 2014 was 7.9% [8].

Early clinical diagnosis of SFTSV infection is crucial for controlling the spread of SFTS. Generally, infection is confirmed via detection of viral genome or virus-specific antibodies in the patient’s blood or serum [9]. Several SFTSV RNA detection kits are currently available [10–12]. However, no references are available for these commercial kits. In this study we established a national reference for SFTSV RNA diagnostic kits refer to the methods of other references [13–17].

## Methods

### Viruses

Six SFTSV strains (AH12, HN1, JS3, LN3, SD4, and HB29 isolated from Anhui, Henan, Jiangsu, Shandong, and Hubei, respectively) were provided by the National Vaccine & Serum Institute (Beijing, China). Japanese encephalitis virus (JEV) SA14-14-2, dengue virus (DENV) Ban18 and yellow fever virus (YFV) 17D were provided by Arbovirus Vaccine Group, National Institutes for Food and Drug Control (NIFDC). Inactivated hemorrhagic fever with renal syndrome virus (HFRSV) and tick-borne encephalitis virus (TBEV) Senzhang strain was provided by Changchun Institute of Biological Products Co., Ltd.

### Materials

Nucleic acid detection kits for SFTSV (PCR-fluorescence probe techniques) were obtained from SinoMD Gene (Beijing, China) and DaAn Gene (Guangzhou, China). An RNA extraction QIAamp Viral RNA Mini Kit (cat 52904) was obtained from Qiagen (Hilden, Germany). Reverse transcriptase was purchased from Promega (Madison, WI, USA), and La Taq was purchased from Takara (Shiga, Japan).

### Selection of references

Four kinds of references (positive, negative, sensitivity and accuracy) were investigated.

#### Positive references

**Fig. 1:**
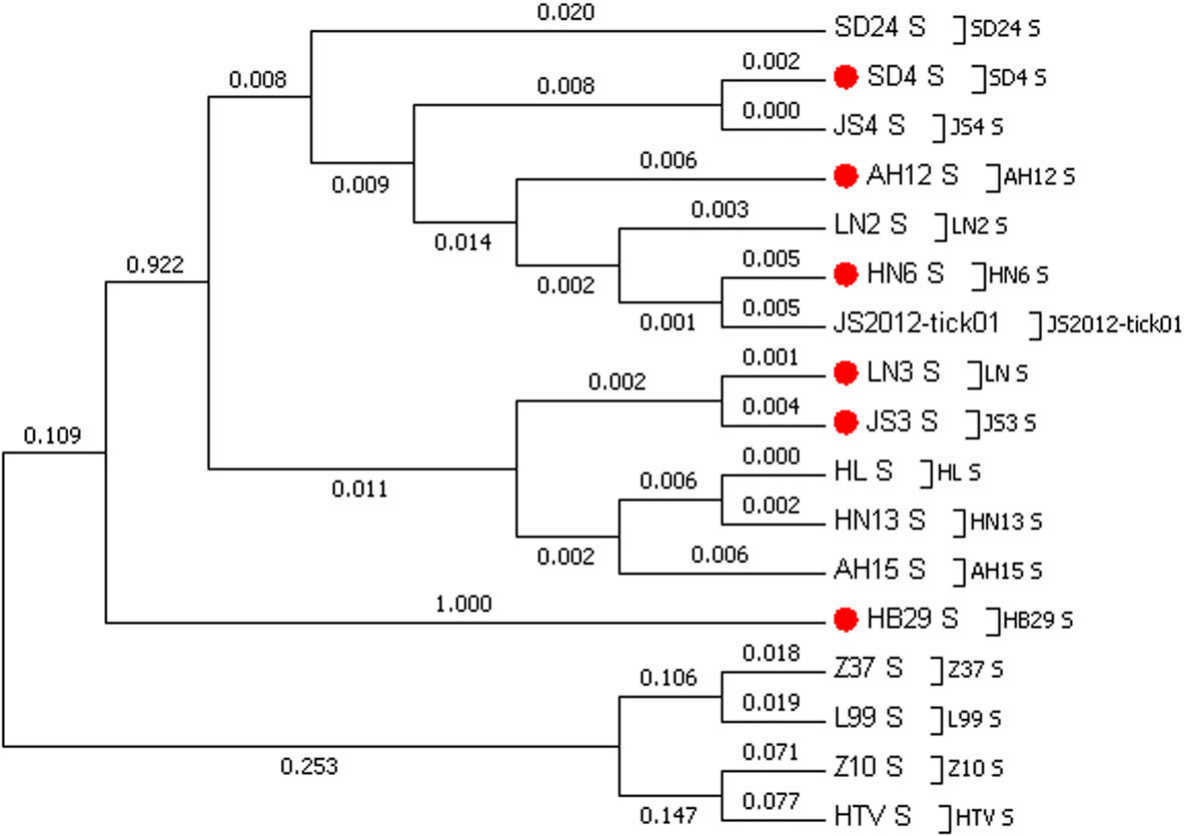
The phylogenetic tree of SFTSV based on S segment. The GenBank number of each virus was listed as follow, SD24 (HM802205.1), SD4 (HM802204.1), JS4 (HQ141606.1), AH12 (HQ141591), LN2 (HQ141609.1), HN6 (HQ141597.1), JS2012-tick01 (KC473542.1), LN3 (HQ141612.1), JS3 (HQ141603), HL (KF791952.1), HN13 (HQ141600.1), AH15 (HQ141594.1), HB29 (HM745932), Z37 (AF187082.1), L99 (AF288299.1), Z10 (EF533944.1), HTV (U37768.1). *Red dots* indicate the strains selected for references

References should be broad-spectrum and representative. So the phylogenetic tree of SFTSV, based on the S segment, was first draw using MEGA 6.0 (Fig. 1). To represent a range of epidemic regions and disease severities, six SFTSV strains AH12, HN1, JS3, LN3, SD4, and HB29, isolated from Anhui, Henan, Jiangsu, Shandong, and Hubei, respectively, were used as positive references.

#### Negative references

Five arthropod-borne or blood-borne viruses (JEV A14-14-2 strain, DENV Ban18 strain, YFV 17D strain, HFRSV L99 strain and TBEV Senzhang strain), which cause similar clinical manifestations to SFTSV infection, including fever, headache, disturbance of consciousness, convulsions, bleeding and kidney damage, meningeal irritation and paralysis, were selected as negative references to evaluate the kit specify [18–22].

The earliest isolated and extensively investigated SFTSV strain HB29 was used as references for sensitivity and accuracy.

### Preparation of the references

Six SFTSV strains AH12, HN1, JS3, LN3, SD4, and HB29 were amplified. Virus titers were determined before inactivation using the cytopathogenic effect assay (CPE). JEV A14-14-2 strain, DENV Ban18 strain and YFV 17D strain was amplified in BHK 21 cells, and the titers were determined using a CPE method. HFRSV L99 strain and TBEV Senzhang strain were provided by Changchun Institute of Biological Products. All viruses were inactivated using formaldehyde (1:1000) at 37 °C for 24 h and stored in −80 °C for use.

### Confirmation of the references

#### Sequence confirmation of the references

The S gene represents a conserved region of the SFTSV genome [3]. The whole genome of JEV is conserved [23]. The NS1 gene represents a conserved region of DENV [24]. The S gene represents a conserved region of HFRSV [25]. 3′ UTR is the conserved region of YFV [21]. 5′UTR is the conserved region of TBEV [22]. The specific primers for the conserved region of each virus were designed using DNAMan (Lynnon, San Ramon, CA, USA).

Virus RNA genome was extracted using QIAamp Viral RNA Mini Kit and then reverse transcribed into cDNA. The cDNA was used as template for PCR in a total volume of 50 μL (LA Taq, 0.2 μL, Buffer 5 μL, dNTP 4 μL, cDNA template 2 μL, primers 2 μL and ddH_2_O 37 μL) using the following procedures: denaturation at 94 °C for 2 min; 30 cycles of 94 °C for 30 s, 56 °C for 30 s, 72 °C for 30 s, and finally extension at 72 °C for 5 min. The PCR products were confirmed by sequencing (Invitrogen, Carlsbad, CA, USA).

#### Confirmation by quantitative PCR (q-PCR)

Specific primers and probe was synthesized for S gene of SFTSV.

SFTSVF: 5′GGGTCCCTGAAGGAGTTGTAAA3′

SFTSVR: 5′TGCCTTCACCAAGACTATCAATGT3′,

SFTSVP: FAM-TTCTGTCTTGCTGGCTCCGCGC-BHQ1

References were quantified using specific primers and probes, SFTSV nucleic acid detection kits (PCR-fluorescence probe techniques) from SinoMD Gene, and DaAn Gene. The reaction system for specific primers and probes were: 0.1 μL LA Taq, 2.5 μL reaction buffer, 2 μL dNTP, 5 μL template, 2 μL primes, 1 μL probe, and 12.4 μL ddH_2_O. The q-PCR procedure was as follows: denaturation at 94 °C for 10 min; 40 cycles of 94 °C for 30s, 56 °C for 30s. The reaction system for the SinoMD kit was: SFTSV PCR buffer 12.5 μL, SFTSV primers and probe mixture 2.5 μL, SFTSV internal template 1.0 μL, DEPC H_2_O 4.0 μL, template 5.0 μL. The q-PCR procedure was as follows: 45 °C for 15 min, 94 °C for 5 min; 40 cycles of 94 °C for 15 s, 60 °C for 35 s; 25 °C for 1 min. The reaction system for the DaAn kit was: SFTSV reaction buffer A 17 μL, SFTSV reaction buffer B 3 μL, template, 5 μL. The q-PCR procedure was as follows: 50 °C for 15 min, 94 °C for 15 min; 45 cycles of 94 °C for 15 s, 55 °C for 45 s.

#### Confirmation of sensitivity

The extensively investigated SFTSV strain HB29 was used as a reference for sensitivity testing. First, the recombinant plasmid containing the conserved S gene pMD18-TS was constructed, serial diluted 1:10 and then used to establish a standard curve. 4.83 × 10^6^copies/mL of SFTSV HB29 was defined as 10^6^ U/mL. Ten-fold serial dilutions of SFTSV HB29 (10^5^ U/mL, 10^4^ U/mL, 10^3^ U/mL, 10^2^ U/mL, 10 U/mL) were used as sensitivity references and labeled S1-S5. The lowest detection limit of the reference was assessed using SinoMD kit and DaAn kit for 4 times.

#### Confirmation of accuracy

The reference (10^4^ U/mL (S3) SFTSV HB29) was detected using the SinoMD and DaAn kits for 10 times and the coefficient of variation (CV) of each Ct was calculated and defined as the accuracy of the reference.

### Stability of the references

Three sets of references stored at −70 °C were randomly selected. One set was placed at 37 °C with 60–80% relative humidity for 8 days, one at room temperature with 20–50% relative humidity for 14 days, and one at −70 °C for 8 months. The stability of these references was determined using the detection kits.

The references stored at −70 °C were subjected to 3 cycles of freeze-thaw, then nucleic acid detection was performed to determine the effect of freeze-thaw treatment.

### Collaborative calibration of the references

According to the Program, Protocol, Standard Operating Procedure of Collaborative Calibration approved by the National Administrative Committee for Certified Reference Materials, and unified original experimental record, the collaborative calibration was completed by the Second Group for in vitro Diagnostics of NIFDC, Arbovirus Vaccine Group of NIFDC and DaAn Gene Co. Ltd.

### Statistical analysis

Statistical analysis was performed using SAS 9.1 (Raleigh, NC, USA), and means are presented. *T* test was used to analyze the stability in each treatment groups. A *p* value < 0.05 was considered to indicate statistical significance.

## Results

### Preparation of the references

Using the CPE method, the titer of SFTSV ranged from 6.8 to 8.3 lg CCID_50_/mL, the titers of the JEV A14-14-2 strain, DV Ban18 strain and YFV 17D strain were 5.56 lg PFU/mL, 5.40 lg PFU/mL and 6.09 l g PFU/mL, respectively. The titers of the provided HFRSV L99 strain and TBEV Senzhang strain were 7.3 lg CCID_50_/mL and 8.0 lg LD_50_/mL, respectively.

### Specificity of the references

Sequencing of these 6 positive and 5 negative references indicated that all conserved virus genes or genomes (S gene of SFTSV, whole genome of JEV, NS1 gene of, S gene of HFRSV, 3′ UTR of YFV and 5′UTR of TBEV) shared 99% homology with genes from the same or similar virus strains.

**Fig. 2:**
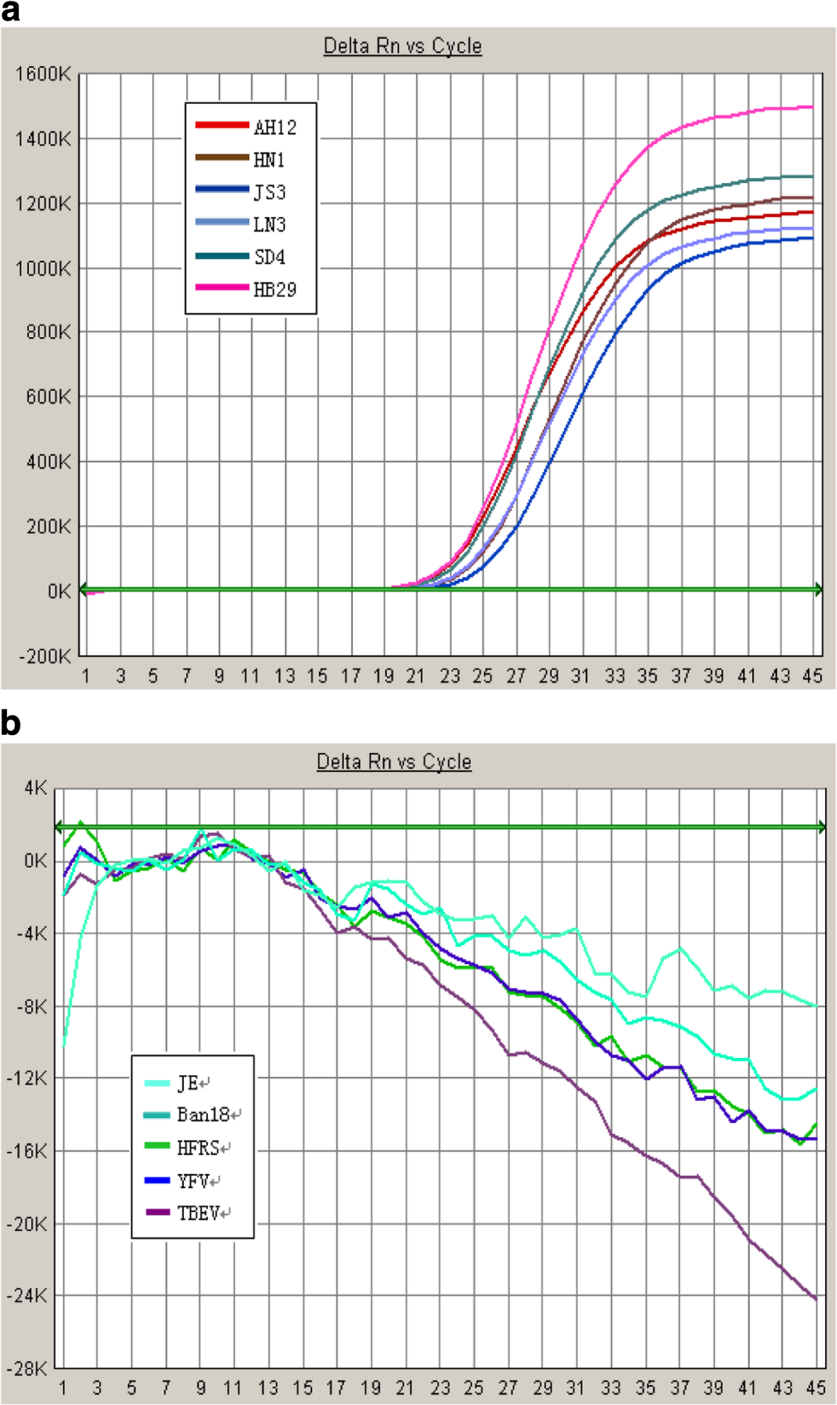
q-PCR reaction curve of positive references (**a**) and negative references (**b**)

Next these positive and negative references were applied for q-PCR using SFTSV specific primers and probes, and kits from SinoMD and DaAn gene. The positive Ct value was defined as less than 35 for specific primers and probes, and less than 37 for the two kits according to the manufacturers’ protocol. The Ct values of 6 positive references were all less than 35, with typical S curves for specific primers and probes as well as two commercial kits (Table 1, Fig. 2a). No typical S curve was observed in five negative samples (Fig. 2b), so no Ct values were given. Our results indicated that these positive and negative references were highly specific and accurate.

**Table 1:** q-PCR results for specificity of references

Strains	Ct value					
	Specific primers and probe		SinoMD kit		DaAn kit	
	Exp 1	Exp 2	Exp 1	Exp 2	Exp 1	Exp 2
AH12	22.89	22.84	24.69	26.50	20.72	18.60
HN1	24.05	23.53	24.96	26.04	20.91	20.15
JS3	25.01	25.26	25.01	25.50	21.94	20.84
LN3	24.29	23.96	24.26	22.61	21.26	20.04
SD4	24.09	24.05	26.90	29.86	21.42	19.27
HB29	23.68	22.99	25.30	23.12	20.44	18.87
Positive	21.89	21.57	17.29	18.99	23.49	19.77
Negative	—	—	—	—	—	—

*Exp* experiment

### Sensitivity of the references

**Fig. 3:**
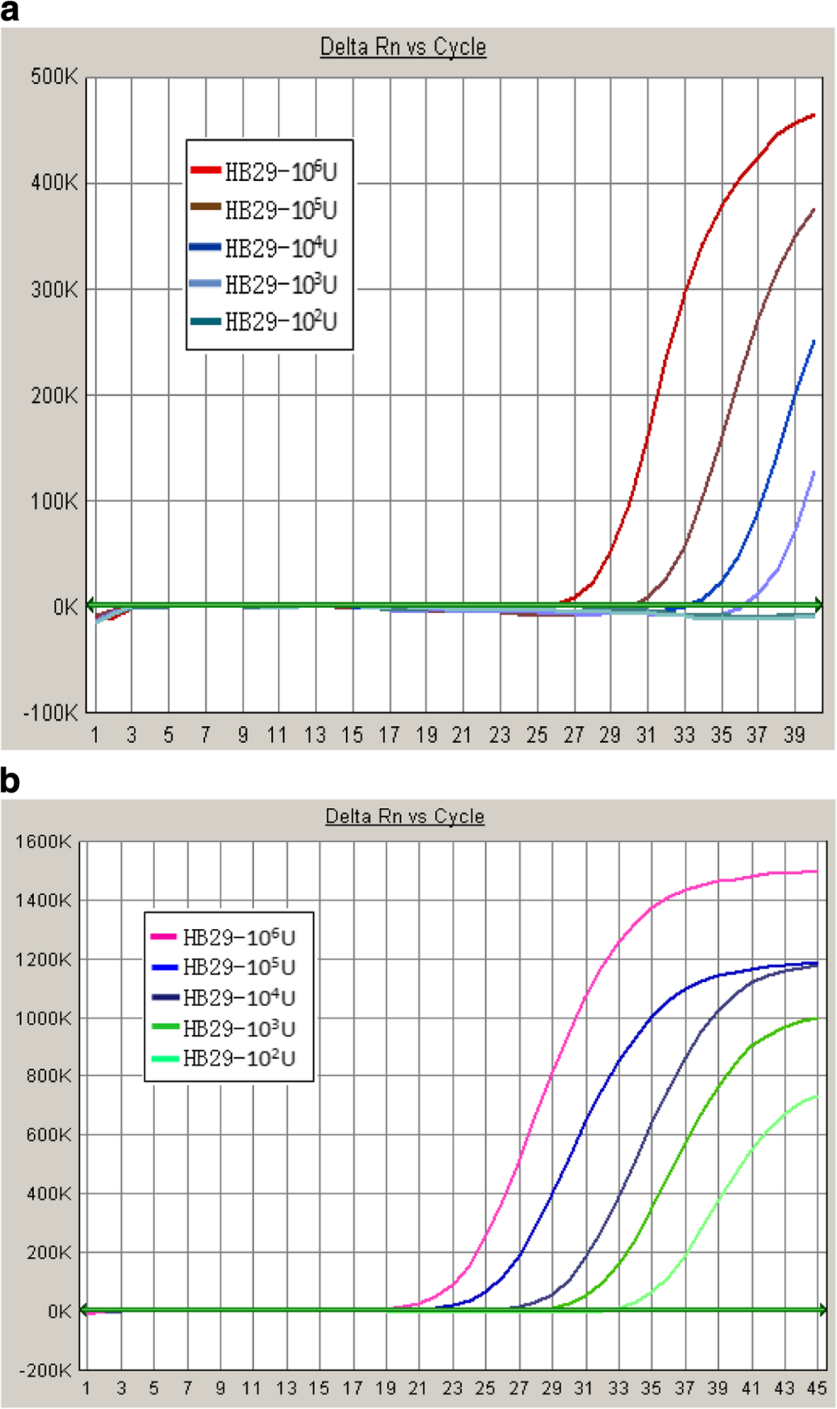
q-PCR reaction curve of sensitivity reference using SinoMD kit (**a**) and DaAn kit (**b**)

The recombinant plasmid pMD18-TS for standard curve ranged from 2.01 × 10^12^ copies/mL to 2.01 × 10^4^ copies/mL in ten-fold dilutions. The sensitivity reference SFTSV HB29 strain was used at 4.83 × 10^6^ copies/mL, serially diluted 1:10 five types (S1-S5), and detected using two commercial kits. As shown in Table 2, the detection limit of the SinoMD kit was S3 with 10^3^ U/mL virus, while the detection limit of the DaAn kit was S4 with 10^2^ U/mL virus. Although S5 with 10 U/mL virus can be detected using the DaAn kit in 3 of 4 times, the Ct value and dilution did not have a linear relationship. The detection limit was still S4 with 10^2^ U/mL virus. The q-PCR reaction curves of sensitivity reference are shown in (Fig. 3a and 3b).

**Table 2:** q-PCR results for sensitivity of reference

HB29 strain	Ct value							
	SinoMD kit				DaAn kit			
	Exp 1	Exp 2	Exp 3	Exp 4	Exp 1	Exp 2	Exp 3	Exp 4
S1 (10^5^ U/mL)	30.03	27.35	29.40	30.23	21.42	22.48	26.37	24.11
S2 (10^4^ U/mL)	32.21	33.16	32.74	33.27	26.05	24.84	28.86	26.69
S3 (10^3^ U/mL)	37.07	34.04	35.60	36.19	28.28	28.32	30.75	28.13
S4 (10^2^ U/mL)	—	37.36	—	—	32.20	29.46	31.79	31.08
S5 (10 U/mL)	—	—	—	—	—	32.37	32.06	31.17
Positive	17.59	16.75	17.84	19.07	19.77	23.49	20.69	20.00
Negative	—	—	—	—	—	—	—	—

*Exp* experiment

The virus titer in the serum of patients with SFTSV infection is reported to usually range between 10^2^ and 10^8^ copies/mL [26–29], so to be clinically useful a reference concentration of 10^2^ U/mL must be detectable by commercial kits.

### Repeatability of the references

**Fig. 4:**
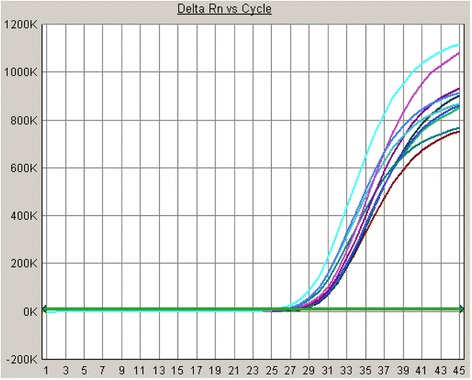
q-PCR reaction curve of accuracy for reference

To evaluate the repeatability of the accuracy reference, a sample containing 10^4^ U/mL (S3) SFTSV HB29 was detected using the SinoMD and DaAn kits in five independent experiments including two duplicates each. As shown in Table 3, the SinoMD kit CV of Ct was 3.98 and 3.16%, while the DaAn kit CV of Ct was 3.04 and 4.55%. So the CV of Ct for this accuracy reference was determined as less than 5%. See (Fig. 4) for q-PCR reaction curve of accuracy for reference.

**Table 3:** q-PCR results for accuracy of reference

References	Ct value			
	SinoMD kit		DaAn kit	
	Exp 1	Exp 2	Exp 1	Exp 2
R1	33.27	33.80	26.05	27.47
R2	34.19	34.98	27.63	27.08
R3	30.72	33.10	27.67	26.22
R4	31.24	33.03	27.38	28.15
R5	32.75	36.36	26.26	27.12
R6	32.19	34.00	26.14	25.56
R7	31.85	33.34	26.11	25.67
R8	32.62	33.93	25.29	24.84
R9	34.43	32.74	27.34	28.03
R10	30.90	34.18	26.89	25.09
SD	1.29	1.07	0.81	1.21
Average	32.42	33.95	26.68	26.52
CV (%)	3.98	3.16	3.05	4.55

*Exp* experiment, *R1-R10* repeat 1-10, *SD* standard deviation, *CV* coefficient of variation

### Stability of all references

**Fig. 5:**
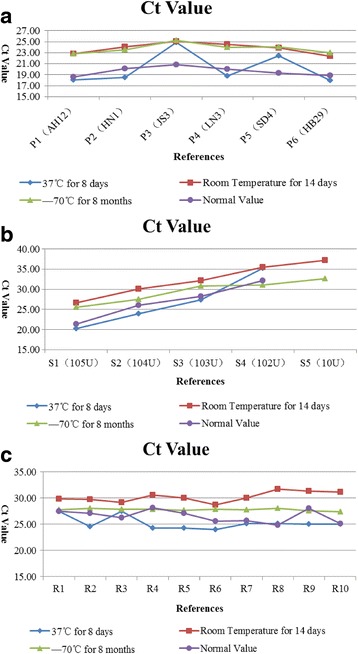
q-PCR results for stability of the references under different treatments. **a** Positive references; **b** Sensitivity references; **c** Accuracy references

The appearance of the references was not altered by storage at 37 °C for 8 days, and at room temperature with 20–50% for 14 days, or at −70 °C for 8 months. All references appeared to be transparent liquids with no turbidity or precipitation. When these references were subjected to q-PCR with specific primers, treated positive references were positive, while all treated negative references were negative, without typical S curves or Ct values higher than 37. The detection limits of all treated sensitivity references were 10^2^ U/mL virus and the CV values of Ct for all treated accuracy references were less than 5% (Table 4, Fig. 5a, 5b and 5c).

**Table 4:** q-PCR results for stability of reference in different storage conditions

References		Ct value			Normal value
		37 °C for 8 days	Room temperature for 14 days	−70 °C for 8 months	
Positive	P1 (AH12)	18.02	22.81	22.84	18.60
	P2 (HN1)	18.49	24.02	23.53	20.15
	P3 (JS3)	24.83	25.07	25.26	20.84
	P4 (LN3)	18.80	24.52	23.96	20.04
	P5 (SD4)	22.41	23.88	24.05	19.27
	P6 (HB29)	17.92	22.35	22.99	18.87
Sensitivity	S1 (105U)	20.20	26.69	25.51	21.42
	S2 (104U)	23.99	30.03	27.46	26.05
	S3 (103U)	27.40	32.23	30.78	28.28
	S4 (102U)	35.29	35.54	31.08	32.20
	S5 (10U)	—	37.23	32.68	—
Accuracy	R1	27.50	29.87	27.77	27.47
	R2	24.56	29.78	28.08	27.08
	R3	27.50	29.21	27.89	26.22
	R4	24.30	30.61	27.89	28.15
	R5	24.27	30.04	27.63	27.12
	R6	23.99	28.70	27.85	25.56
	R7	25.09	30.03	27.81	25.67
	R8	25.11	31.76	28.01	24.84
	R9	25.01	31.39	27.60	28.03
	R10	25.06	31.16	27.37	25.09
	SD	1.26	0.97	0.21	1.21
	Average	25.24	30.26	27.79	26.52
	CV	5.00%	3.20%	0.75%	4.55%

The references were subjected to 3 cycles of freeze-thaw treatment and after each cycle tested using q-PCR. The accuracy reference CV values of Ct did not differ between the three cycles (Tables 5, *P* > 0.05). All these data suggest that all references exhibit good stability to temperature.

**Table 5:** q-PCR results for stability of the references subjected to freeze-thraw

Reference	Ct value		
	First F-T	Second F-T	Third F-T
HB29 strain	25.49	26.23	27.31
	26.29	25.80	26.58
	26.10	26.28	25.80
Average	25.96	26.10	26.56
HB29 strain at 1:100	33.27	31.85	34.43
	34.19	32.62	30.90
	30.72	32.75	33.48
	31.24	32.19	33.00
Average	32.36	32.35	32.95

*F-T* freeze-thaw

#### *Calibration* o*f the references*

Calibration of the references is described in Table 6. In three independent laboratories the positive rate of positive references and negative rate of negative references were all 100%. The detection limit of the sensitivity reference was 10^2^ U/mL. The CV of the accuracy reference ranged from 0.45 to 4.55%, which were all less than 5%.

**Table 6:** Calibration of the references

Labs	Exp	Positive coincidence rate	Negative coincidence rate	Sensitivity (10^2^ U/mL)	Accuracy (CV)
Second group for in vitro diagnostics of NIFDC	1	100%	100%	Detected	3.49%
	2	100%	100%	Detected	0.45%
	3	100%	100%	Detected	3.80%
	Average	100%	100%	Detected	2.58%
Arbovirus vaccine group of NIFDC	1	100%	100%	Detected	3.98%
	2	100%	100%	Detected	3.16%
	3	100%	100%	Detected	3.05%
	4	100%	100%	Detected	4.55%
	Average	100%	100%	Detected	3.69%
DaAn gene Co. Ltd	1	100%	100%	Detected	1.97%
	2	100%	100%	Detected	1.53%
	3	100%	100%	Detected	0.56%
	4	100%	100%	Detected	1.44%
	5	100%	100%	Detected	1.19%
	Average	100%	100%	Detected	1.34%

## Discussion

SFTS caused by SFTSV infection was first emerged in China in 2009 [3], followed by reports from other Asian countries. At present no effective therapies or vaccines are available, and SFTS is associated with high rates of fatality [1, 5, 30]. In addition to reducing rates of tick bite, early diagnosis and treatment are important. Hence many research institutes and companies are developing diagnostic kit for SFTSV based on the detection of virus genome using q-PCR. However, no references are yet available to control the quantity of these kits. Thus in this study we established national references that allow specific, sensitive and accurate detection of the SFTSV genome.

When establishing reference standards, the references should be represent a broad-spectrum of relevant viruses [31]. In 2007, the first SFTS case in China was reported in Henan Province, and since cases have been reported in Shandong, Hubei, Anhui, Jiangsu, and Niaoning [1]. Thus we chose viruses from these regions to use as the reference strains. The severity of the disease caused by these virus strains also should be considered. We drew a phylogenetic tree based on S segment and selected six representative SFTSV strains, AH12, HN1, JS3, LN3, SD4, and HB29, isolated from Anhui, Henan, Jiangsu, Liaoning, Shandong, Hubei respectively, as positive references. When selecting negative references, HFRSV was chosen because of the similar structure and family to SFTSV [32]. JEV, DenV, YFV and TBEV were chosen as they cause similar clinical symptoms and are transmitted by arthropods or blood, like SFTSV [18–22]. Before establishing references, conserved regions of these 11 viruses were amplified using specific primers and sequenced. Then q-PCR was used to detect the specificity and accuracy of positive and negative references.

When selecting sensitive and accurate references, the earliest identified and extensively studied HB29 strain was chosen. The serial dilution method was used to determine the detection limit of the reference. Through collaborative calibration by three labs, the detection limit was set as 10^2^ U/mL, as SFTSV infection is reported to cause titers of 10^2^-10^8^copies/mL in the serum [26–29]. The CVs of Ct were all less than 5%, indicating acceptable accuracy.

We also investigated the capacity of the references to withstand freeze-thaw and conditions typical of storage, transportation and application, as previously reported [33]. Storage at room temperature, 37 °C and several freeze-thaw cycles did not alter the specificity, accuracy, and sensitivity of the references, indicating high stability.

## Conclusions

The national reference had been established successfully and proved good accuracy, high specificity, sensitivity and stability. It means that the reference will be widely used in the quality control of the similar commercial SFTSV diagnostic kits and guarantee their effectiveness, thus more SFTSV infection can be diagnosed rapidly and the targeted prevention and control measures will be developed.

## Additional file

Additional file 1: The q-PCR results and phylogenetic tree of SFTSV study. (RAR 2243 kb)
